# A lightweight deep learning framework for tea cultivar identification based on leaf images

**DOI:** 10.3389/fpls.2026.1863075

**Published:** 2026-08-20

**Authors:** Menghan He, Guanjun Chen, Jianzhao Wang, Dandan Tang, Qinling Liu, Xiong Xiong, Liqiang Tan

**Affiliations:** 1 Tea Resources Utilization and Quality Testing Key Laboratory of Sichuan Province, Sichuan Agricultural University, Chengdu, China; 2 College of Horticulture, Sichuan Agricultural University, Chengdu, China; 3 College of Information Engineering, Sichuan Agricultural University, Yucheng District, Ya’an, China

**Keywords:** tea plant cultivars, cultivar identification, leaf image analysis, tea cultivar identification, deep learning

## Abstract

Accurate tea cultivar identification is a long-standing challenge in tea plant propagation and cultivation. Traditional cultivar identification based on morphological observation relies heavily on expert experience, leading to strong subjectivity; while DNA fingerprinting-based approaches are time-consuming and costly, restricting their large-scale application. This study attempts to construct a deep learning- based on leaf morphological and visual characteristics. We collected an original dataset comprising 11,404 leaf images from 10 clonal tea cultivars for model development and evaluation. Comparative experiments were conducted using five models: ResNet50, EfficientNet-B3, MobileNetV3-large, ViT-B16, and ConvNeXt-Tiny. ResNet50 achieved the highest classification accuracy, whereas MobileNetV3-Large provided a better balance between accuracy and computational efficiency and was therefore selected as the baseline model for lightweight optimization. Channel pruning and PTDQ were further applied to compress and optimize MobileNetV3-Large, resulting in a lightweight and efficient tea cultivar identification model. The optimized model achieved a test accuracy of 97.15% on the independent testing set, while reducing the model size from 34.40 MB to 7.67 MB and decreasing inference latency from 42.35 ms to 27.67 ms. Future work will focus on integration into Android-based field applications. This study provides an efficient lightweight solution for practical tea cultivar identification and mobile deployment.

## 1 Introduction

Tea plants are evergreen woody shrubs and tea processed from their leaves is a widely consumed beverage worldwide (Tang et al., 2019; Du et al., 2022) There are many varieties of tea plants and most of them are clonal tea cultivars that propagated through cuttings (Fang et al., 2011). For instance, in China alone, there are over 500 registered clonal tea cultivars, covering more than 80% of Chinese tea gardens (Chen et al., 2012). This has resulted in extensive cultivar diversity and the need for accurate tea cultivar identification.

Different clonal tea cultivars exhibit significant differences in cultivation adaptability, nutritional composition, yield levels, and quality characteristics (Nyabundi et al., 2017; Zaman et al., 2023; Zhao et al., 2024). Market prices for their seedlings and tea products also vary significantly (Akankwasa et al., 2025). The tea seedling market has long been plagued by issues such as impure cultivars, synonyms and homonyms. Therefore, accurate identification of tea cultivars is crucial for regulation the propagation and sale of tea seedlings, also for protecting the rights of breeders, tea farmers, and consumers (Fang et al., 2014; Hu et al., 2023). Traditional tea plant cultivar identification primarily relies on experts manually distinguishing external morphological characteristics based on experience and expertise (Luo et al., 2024). Many studies also employ techniques such as chemical component analysis, SSR molecular markers, and DNA fingerprinting to accurately identify tea cultivars (Wang et al., 2016b; Fang et al., 2017). Although these approaches provide reliable genetic evidence, they generally require laboratory facilities, specialized personnel, and relatively long processing times, limiting their application in rapid field-level identification.

In recent years, computer vision and deep learning approaches have been increasingly applied to crop variety identification. Traditional image-processing approaches based on manually designed color, shape, and texture features have also been explored for crop variety identification, but their performance is often affected by environmental variations and feature engineering requirements. Spectral imaging techniques, particularly hyperspectral and near-infrared imaging, have attracted increasing attention in precision agriculture because they can capture biochemical and structural information beyond conventional RGB images. Recent studies have demonstrated the effectiveness of combining hyperspectral imaging with deep learning and explainable AI for plant disease detection and feature interpretation (Goyal et al., 2025; Malik et al., 2026). However, hyperspectral systems generally require specialized equipment and acquisition procedures, whereas RGB image-based approaches offer a more accessible and cost-effective solution for practical cultivar identification. deep learning has achieved substantial progress in crop variety identification, particularly in image-based automatic recognition, where it demonstrates high accuracy and robustness (Li et al., 2020; Chen et al., 2023). Convolutional neural networks (CNNs), as representative approaches, can automatically extract discriminative features from plant leaves, fruits, and whole-plant morphological characteristics, enabling efficient classification of different varieties (Bouakkaz et al., 2025; Patrício and Rieder, 2018). For example, Jin et al. combined near-infrared hyperspectral imaging with a CNN model to identify rice seed varieties, achieving classification accuracy above 95% (Jin et al., 2022). Meanwhile, Yang et al. developed a CNN model based on the Keras framework and achieved an average recognition accuracy of 95.49% for three maize varieties (Yang et al., 2023), while Dong et al. proposed a feature extraction model for Camellia oleifera leaves and classified 118 varieties under natural lighting conditions, achieving an accuracy of 93.7% (Dong et al., 2024). These studies demonstrate the effectiveness of deep learning for crop variety identification.

Recent studies have further demonstrated the potential of deep learning in tea production and cultivar identification. Zhang et al. developed a hybrid framework combining convolutional neural networks and machine learning algorithms for tea cultivar discrimination using canopy images, achieving high classification performance under field conditions. This study highlighted the feasibility of deep learning-based visual approaches for intelligent tea cultivar analysis (Zhang et al., 2026). Furthermore, Zhang et al. investigated the influence of dataset construction and training strategies on tea variety identification during the harvest period, demonstrating that optimized dataset organization and training schemes could effectively improve model performance (Zhang et al., 2025a). These studies indicate that deep learning has considerable potential for tea cultivar identification; however, existing approaches mainly focus on canopy-level images, while leaf-level lightweight identification suitable for mobile deployment remains insufficiently explored.

Tea leaves contain cultivar-related morphological and visual features, including leaf shape, vein architecture, texture patterns, and pigmentation, which are partly determined by genetic background and therefore provide valuable information for cultivar discrimination (Li et al., 2022a). Compared with major crops, tea cultivar identification based on leaf images remains challenging under practical field conditions due to variations in illumination, leaf age, growth stage, and cultivation environment. Therefore, developing a deep learning-based approach for tea cultivar identification using leaf images has significant potential for rapid and practical applications (Wang et al., 2024).

**Figure 1:**
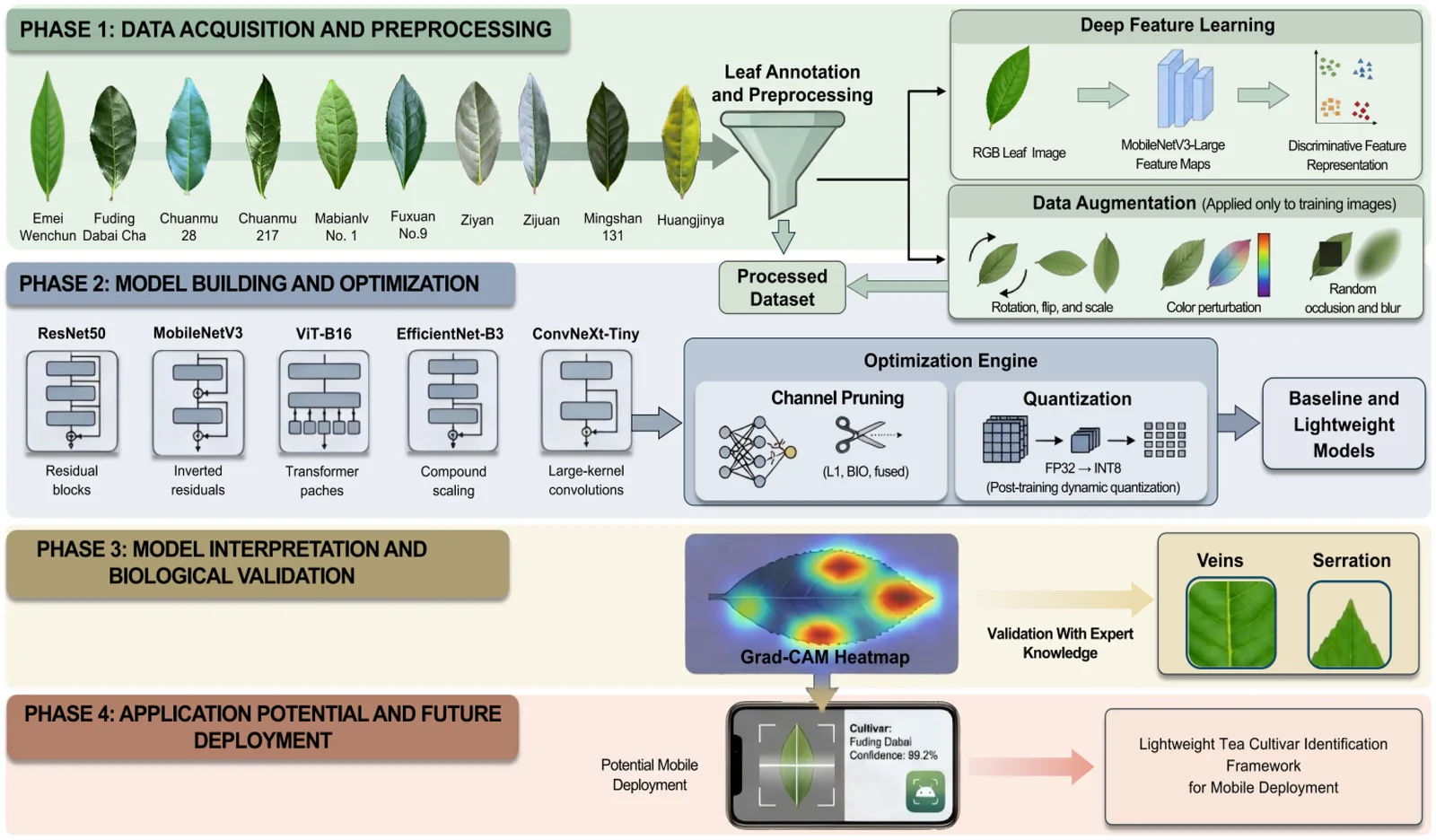
Overall process for tea cultivar identification based on leaf characteristics.

In this study, we collected leaf images from 10 tea cultivars to establish an RGB image dataset. Five CNN and lightweight CNN architectures were compared to select an appropriate baseline model. MobileNetV3-Large, selected based on the balance between classification accuracy and computational efficiency, was further optimized through pruning and PTDQ. The proposed lightweight framework provides an efficient solution for tea cultivar identification and has potential for future mobile-assisted field applications (**Figure 1**).

## 2 Materials and methods

### 2.1 Tea plant materials

Ten tea cultivars were selected for the analysis, including Emei Wenchun, Fuding Dabai Cha, Chuanmu 217, Chuanmu 28, Mabianlv No. 1, Fuxuan No. 9, Mingshan 131, Ziyan, Zijuan, and Huangjinya. These cultivars represent diverse genetic backgrounds and exhibit differences in leaf morphology, color, texture, and agronomic characteristics (**Supplementary Figure 1**; **Table 1**). The leaf samples were collected during the summer growth period from June to July 2025 through four independent acquisition sessions. Each acquisition session was conducted between 13:00 and 16:00 to reduce variations in illumination conditions. The ambient temperature during image acquisition ranged from approximately 20 to 35 °C. Tea leaf images were collected using two acquisition modes: direct imaging of leaves attached to tea plants and imaging of detached leaves after collection. The combination of these two acquisition conditions was designed to introduce realistic variations in field image acquisition, including differences in leaf orientation, background complexity, illumination, and camera distance. For detached-leaf acquisition, fully expanded and healthy mature leaves without visible disease symptoms, insect damage, or mechanical injury were manually collected and photographed immediately after detachment. For *in situ* acquisition, leaves were directly photographed on tea plants under field conditions. Images from both acquisition modes were included in the dataset to improve the robustness and practical applicability of the proposed model. Images were captured using multiple smartphone cameras, including Huawei, Xiaomi, and Apple devices, with a minimum resolution of 4032 × 3024 pixels. The images were collected under field environments with variations in illumination intensity, shadow distribution, background complexity, and leaf orientation, thereby introducing realistic variability for model evaluation. During each acquisition session, leaves from different cultivars and individual plants were collected following the same imaging protocol. However, images acquired within the same session were assigned to only one dataset subset to avoid potential information leakage.

**Table 1:** Basic information and morphological characteristics of the ten tea cultivars.

Cultivar	Origin	Major agronomic traits	Leaf characteristics
Emei Wenchun	Sichuan	Bush type, medium-leaf category, extra-early maturing.	Leaves are medium to small, oblong-elliptical in shape. Leaf color is deep green with a glossy sheen. Leaf surface is slightly convex, with a slight inward fold along the midrib. Leaf texture is soft, and leaf teeth are relatively sharp.
Fuding Dabai Cha	Fujian	Small tree type, medium-sized leaves, early maturing.	Leaves are medium-sized and elliptical in shape. Leaf color is yellowish-green (glossy), with a convex surface and flat texture. The leaf is relatively thick yet soft, featuring prominent lateral veins.
Chuanmu 217	Sichuan	Bush type, medium-leaf category, with larger leaves and early maturity.	Leaves are relatively large, oblong-elliptic or lanceolate in shape. Leaf color is green, with a flat or slightly convex surface. The leaf texture is soft, exhibiting strong tenderness retention.
Chuanmu 28	Sichuan	Bush type, medium-leaf category, extra-early maturing.	Leaves are medium-sized and elliptical. Leaf color is deep green with a convex surface. Leaf texture is thick and tender. Buds and leaves are yellow-green with moderate pubescence.
Mabianlv No. 1	Sichuan	Small-tree type, large-leaf category, medium-season maturing.	Large leaves, oblong or elliptical in shape. Deep green color, flat surface, relatively thick texture, with distinct wavy margins.
Fuxuan No. 9	Fujian	Small-tree type, medium-leaf category, extra-early maturing.	Medium-sized leaves, nearly elliptical in shape. Green color, convex surface, slightly inward-curved blade, soft texture, plump buds and leaves.
Mingshan 131	Sichuan	Bush type, medium-leaf category, early-maturing. Leaves are medium-sized and elliptical.	Leaf color is deep green with a slightly raised surface. The leaf blade lies flat or is slightly inward-curved, with a soft texture. Buds and young leaves are green with a hint of purple.
Ziyan	Zhejiang	Shrub type, medium-leaf category, early-ripening.	Spring shoots, leaves, and stems exhibit purplish-red hues, with mature leaves transitioning to deep green with purple undertones. Leaves are oblong-elliptical, slightly convex, and thin in texture.
Zijuan	Yunnan	Small shrub type, medium-leaf category, late-maturing.	Consistently purple-red year-round (buds, leaves, stems, petioles). Large, lanceolate leaves with flat surfaces and thin texture; sharp, dense serrations.
Huangjingya	Zhejiang	Shrub type, medium-leaf category, late-season cultivar.	New shoots maintain golden yellow coloration year-round, with leaf hue varying in intensity based on light exposure. Leaves are long-elliptical with slightly raised surfaces; thin and supple texture.

### 2.2 Dataset construction

A total of 11,404 leaf images from 10 tea cultivars were collected for model development. To avoid potential information leakage caused by visually similar images, dataset partitioning was performed based on acquisition sessions rather than individual images. Images obtained during the same acquisition session were assigned exclusively to the training, validation, or testing subset.

The original dataset was first divided into training, validation, and testing subsets (**Table 2**). The training, validation, and testing sets contained 6,797, 2,236, and 2,371 original images, respectively. After dataset partitioning, augmentation operations were applied only to the training subset, generating 2,088 additional training samples. Therefore, the final training dataset contained 8,885 images, whereas the validation and testing sets remained unchanged.

**Table 2:** Dataset composition and distribution among training, validation, and testing subset.

Cultivar	Original images	Train (raw)	Augmented images	Train (after augmentation)	Val	Test
Fuding Dabai Cha	1115	664	259	923	220	231
Chuanmu 217	1083	646	155	801	213	224
Chuanmu 28	1099	653	297	950	215	231
Emei Wenchun	1112	663	243	906	218	231
Fuxuan No. 9	1071	639	84	723	211	221
Huangjingya	1069	638	167	805	209	222
Zijuan	1040	619	321	940	202	219
Ziyan	1670	997	159	1156	328	345
Mabianlv No. 1	1075	641	191	832	212	222
Mingshan 131	1070	637	212	849	208	225
**Total**	**11404**	**6797**	**2088**	**8885**	**2236**	**2371**

Bold values represent the total number of images in each subset.

### 2.3 Dataset annotation and partitioning

Images were first filtered to remove blurred, low-quality, and heavily occluded samples. The Labeling annotation tool was used to manually annotate individual tea leaf regions using bounding boxes, and the annotations were saved in Pascal VOC XML format, as shown in **Supplementary Figure 2** (Zhao et al., 2022). The “leaf” label was used only to define the region of interest (ROI) for leaf cropping rather than for training an object detection model. Based on the annotated bounding boxes, leaf regions were cropped to reduce background interference, based on the annotated bounding boxes, the leaf regions were cropped to reduce irrelevant background information and resized to 448 × 448 pixels before being input into the deep learning models for cultivar classification. No pixel-level segmentation or explicit background removal was performed. The same preprocessing procedure was applied to the inference images to maintain consistency between the training and testing stages.

### 2.4 Data preprocessing and augmentation

**Figure 2:**
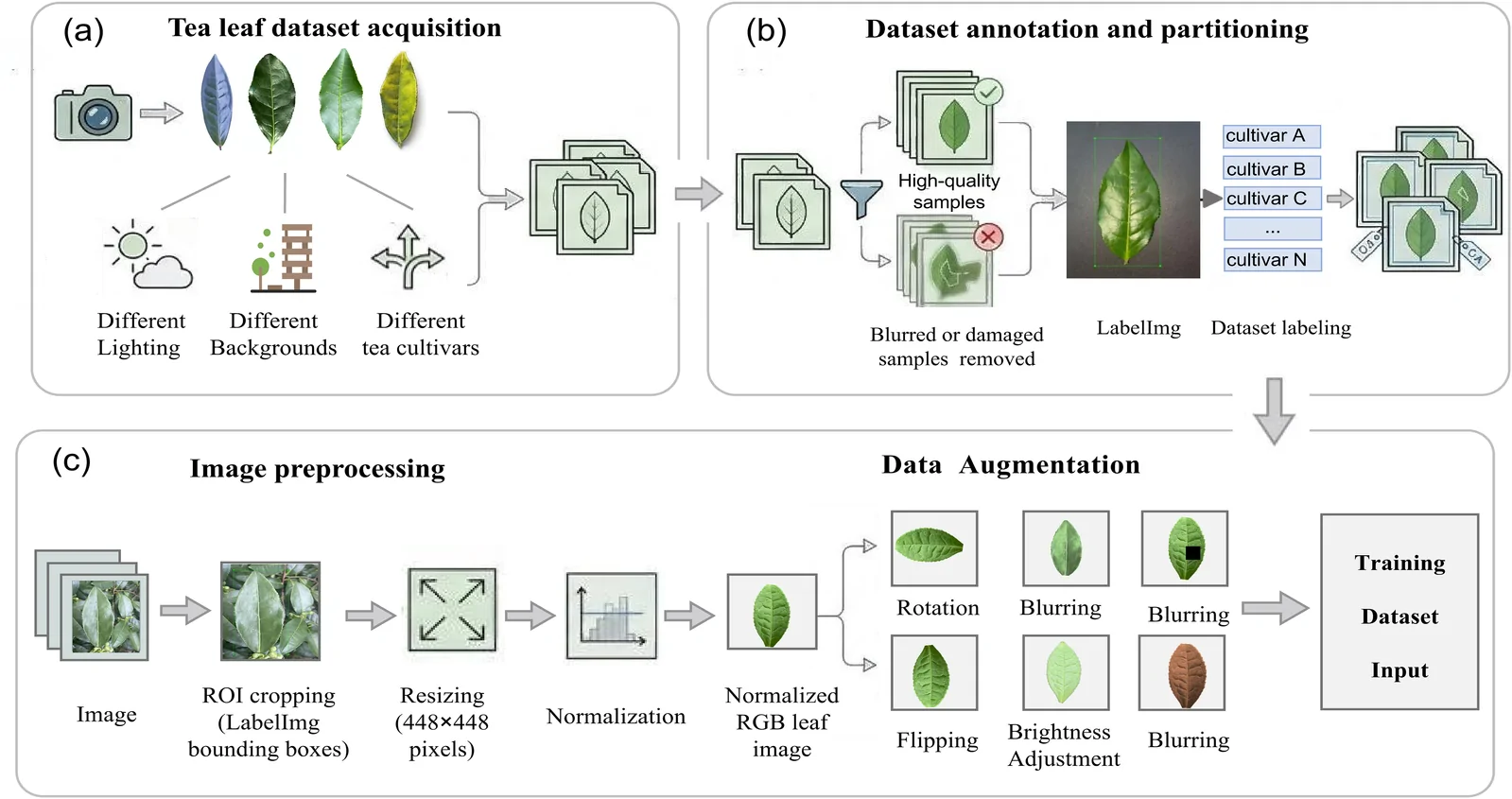
Tea leaf dataset construction, preprocessing, and augmentation pipeline. **(a)** Tea leaf dataset acquisition under different lighting conditions, backgrounds, and tea cultivars; **(b)** dataset annotation and partitioning, including quality control, labeling, and dataset division; **(c)** image preprocessing and augmentation, including ROI cropping, resizing, normalization, and data augmentation.

Prior to model training, leaf images were subjected to standardized preprocessing, including ROI cropping, resizing, and normalization, to reduce irrelevant background information while preserving key morphological characteristics (**Figure 2**). The deep learning models subsequently learned discriminative representations associated with leaf morphology, vein structure, and surface texture directly from the processed RGB images. The final input to all deep learning models was a normalized RGB leaf image with a resolution of 448 × 448 pixels. During training, augmented versions of these images were used as network inputs.

To improve dataset diversity and model robustness under variable acquisition conditions, data augmentation was performed through geometric transformations, appearance variations, and environmental perturbation (Li et al., 2022b). Specifically, random rotation, flipping, and scaling (crop ratio: 0.8–1.0) were applied to introduce geometric diversity. Contrast adjustment, hue shifting, and Gaussian noise addition were employed to simulate variations in illumination and imaging conditions. Furthermore, random occlusion (up to three randomly located regions) and motion blur were introduced to mimic partial obstruction and image degradation encountered during practical image acquisition. After augmentation, 2,088 additional training images were generated, resulting in a final training set of 8,885 images, whereas the validation and testing sets remained unchanged for unbiased performance evaluation.

### 2.5 Selection of representative deep vision backbones and experimental settings

To evaluate the performance and computational efficiency of different visual representation paradigms, five representative deep learning backbones were selected: ResNet50 (Behera et al., 2026), EfficientNet-B3 (Alhichri et al., 2021), MobileNetV3-Large (Yehulu et al., 2025), ViT-B16 (Özben and Güler, 2025), and ConvNeXt-Tiny (Sajid et al., 2026). All input images were resized to 448 × 448 pixels before being fed into the networks. The selected models encompass traditional CNNs, lightweight efficient networks, mobile networks, transformer-based vision models, and modern convolutional networks that fuse convolutional and Transformer design philosophies. Specifically, ResNet50 represents residual learning-based CNN architectures; EfficientNet-B3 achieves an improved accuracy–efficiency trade-off through compound scaling; MobileNetV3-Large is designed for resource-constrained mobile deployment; ViT-B16 introduces pure self-attention-based visual representation learning; and ConvNeXt-Tiny represents a modern CNN architecture incorporating design principles from Transformers.

To ensure a fair comparison, all backbone models were trained and evaluated using identical dataset partitions, preprocessing procedures, augmentation strategies, and evaluation protocols. The dataset partitioning strategy followed the procedure described in Section 2.2. All input images were resized to 448 × 448 pixels to preserve cultivar-related visual patterns in tea leaf images…

All experiments were implemented using PyTorch 1.12.1 with CUDA 11.6 under Python 3.9 and Conda 4.14.0. The experiments were conducted on a workstation equipped with an Intel Xeon Silver 4210R CPU, 128 GB RAM, and an NVIDIA GeForce RTX 3090 GPU with 24 GB memory. All backbone models were initialized using ImageNet-pretrained weights and trained using the same optimization strategy. The Adam optimizer was adopted with an initial learning rate of 2.4 × 10^-^⁴, weight decay of 1 × $10^{-4}$, and batch size of 32. The learning rate was adjusted using a cosine annealing scheduler, and cross-entropy loss was used as the optimization objective. Data augmentation was applied only to the training set. The detailed training hyperparameters and experimental configurations are provided in **Supplementary Table 1**.

### 2.6 Evaluation metrics for multi-class tea cultivar classification

Macro-average metrics were adopted because they assign equal importance to each cultivar category regardless of sample size. To evaluate model classification performance, accuracy, macro-precision, macro-recall, macro-F1 score, and confusion matrices were used as evaluation metrics. The calculation formulas for these evaluation metrics are presented in Equations (1–5). Accuracy measures the proportion of correctly classified samples among all test samples, defined as:.

(1) $$Accuracy=\frac{\sum_{i=1}^{C}TP_{i}}{N}$$

Precision measures the proportion of samples predicted as a given cultivar that actually belong to that cultivar, defined as:

(2) $$Precision_{macro}=\frac{1}{C}\overset{C}{\underset{i=1}{\sum }}\frac{TP_{i}}{TP_{i}+FP_{i}}$$

Recall measures the proportion of actual samples of a given cultivar that are correctly identified, defined as:

(3) $$Recall_{macro}=\frac{1}{C}\overset{C}{\underset{i=1}{\sum }}\frac{TP_{i}}{TP_{i}+FN_{i}}$$

The F1-score is the harmonic mean of precision and recall, defined as:

(4) $$F1_{macro}=\frac{1}{C}\overset{C}{\underset{i=1}{\sum }}\frac{2P_{i}R_{i}}{P_{i}+R_{i}}$$

In the formula, C denotes the number of cultivar classes, N represents the total number of test samples. ${\text{TP}}_{\text{i}}$ ${\text{FP}}_{\text{i}}$ and ${\text{FN}}_{\text{i}}$ denote the numbers of true positive, false positive, and false negative samples for ${\text{class}}_{\text{i}}$, respectively. ${\text{ P}}_{\text{i}}$ and ${\text{R}}_{\text{i}}$ represent the precision and recall of ${\text{class}}_{\text{i}}$, respectively.

The confusion matrix was used to visualize the class-wise classification performance by comparing model predictions with the true labels. For the multi-class classification task, the confusion matrix is represented as a C×C matrix, where the element ${\text{M}}_{\text{ij}}$ denotes the number of samples belonging to the true ${\text{class}}_{\text{i}}$ that are predicted as ${\text{class}}_{\text{j }}$:

(5) $$\left[\begin{array}{cc} TP & FP\\ FN & TN \end{array}\right]$$

### 2.7 Structured channel pruning strategies

To reduce the computational complexity of the selected MobileNetV3-Large baseline, structured channel pruning was applied to its inverted residual blocks. Unlike unstructured pruning, which removes individual weights, structured pruning eliminates entire convolutional channels and therefore produces a compact network architecture that can be directly accelerated on edge devices without requiring sparse-computing libraries. The stem convolution and final classifier were excluded from pruning to preserve the initial feature-extraction capacity and output dimensionality of the network. When an output channel was removed, the corresponding input channels of the subsequent convolutional layer, together with the associated batch-normalization and squeeze-and-excitation (SE) parameters, were adjusted synchronously to maintain dimensional consistency. The detailed pruning configuration is provided in **Supplementary Table 2**.

Three channel-importance estimation strategies were investigated: L1-norm-based pruning, biological feature-guided pruning (BIO), and the proposed FUSED pruning strategy. The L1 criterion evaluates the structural importance of a convolutional channel according to the magnitude of its weights. For output channel c, the L1 importance score was calculated as, the calculation formulas for these evaluation metrics are presented in Equations (6–8):

(6) $$S_{\text{L}1}(c)=\sum_{i}|w_{c,i}|$$

Where ${\text{w}}_{\text{c}}$ denotes the weights of channel c and ${\text{w}}_{\text{i}}$ represents an individual weight element. Channels with smaller L1 scores were considered less important and were removed according to predefined pruning ratios.

To incorporate tea-leaf-specific morphological information into channel evaluation, a biological feature-guided pruning strategy was developed. Biological probes were generated from the original tea-leaf images using an edge-enhancement operation to emphasize cultivar-related structures, particularly leaf veins and boundaries. The generated probes were then passed through the trained baseline model, and the response of each candidate channel was recorded. For output channel c, the BIO importance score was calculated according to the average activation response to the biological probes:

(7) $$S_{\text{BIO}}(c)=\frac{1}{N}\sum_{p=1}^{N}[\frac{1}{H_{c}W_{c}}\overset{{\text{H}}_{\text{c}}}{\underset{\text{x}=1}{\sum }}\overset{{\text{W}}_{\text{c}}}{\underset{\text{y}=1}{\sum }}{\text{A}}_{\text{c}}^{\left(\text{p}\right)}(\text{x},\text{y})]$$

where ${\text{A}}_{\text{c}}^{\left(\text{p}\right)}(\text{x},\text{y})$denotes the activation value of channel c at spatial position (x,y) in response to biological probe p, ${\text{H}}_{\text{c}}$ and ${\text{W}}_{\text{c}}$ are the height and width of the corresponding feature map; and N represents the total number of biological probes. Channels exhibiting stronger average responses to leaf-vein and boundary structures were assigned higher BIO importance scores and were preferentially retained during pruning.

The proposed FUSED strategy integrates three complementary sources of channel importance: structural importance derived from the L1 norm, biological feature sensitivity derived from BIO probes, and model-intrinsic channel importance obtained from the SE attention module. For SE-equipped inverted residual blocks, the SE importance score of channel ccc was obtained by averaging its channel-wise attention coefficients over the probe dataset. Because the three criteria have different numerical ranges, their scores were normalized to [0,1] before fusion. The final channel-importance score was calculated as

(8) $${\text{S}}_{\text{FUSED}}(\text{c})={\text{w}}_{1}{\hat{S}}_{\text{L}1}(\text{c})+{\text{w}}_{2}{\hat{S}}_{\text{BIO}}(\text{c})+{\text{w}}_{3}{\hat{S}}_{\text{SE}}(\text{c})$$

Where ${\hat{S}}_{\text{L}1}(\text{c})$ ${\hat{S}}_{\text{BIO}}(\text{c}),$ and ${\hat{S}}_{\text{SE}}(\text{c})$ represent the normalized importance scores of channel c obtained from L1-norm-based pruning, biological feature-guided pruning, and SE attention, respectively. ${\text{w}}_{1}$ ${\text{w}}_{2}$ and ${\text{w}}_{3}$ are weighting coefficients that determine the contribution of each criterion. Channels were ranked according to the fused importance score, and low-ranked channels were removed according to the predefined pruning ratio. In this study, the weighting coefficients were empirically set as ${\text{w}}_{1}=0.2,{\text{w}}_{2}=0.5{\text{ and w}}_{3}=0.3\;$based on preliminary experiments. The relatively larger weight assigned to the BIO criterion was intended to emphasize channels responsive to tea-leaf morphological characteristics.

For each eligible layer, channels were ranked in ascending order according to the corresponding importance score, and the lowest-ranked channels were removed at a predefined pruning rate. Pruning rates ranging from 10% to 40% were evaluated to investigate the trade-off between classification performance and computational complexity. In the ablation experiments, a fixed pruning rate of 20% was used to ensure that the L1, BIO, L1+BIO, and FUSED variants had identical numbers of parameters and FLOPs.

## 3 Results of backbone comparison and baseline model selection

After channel removal, each pruned model was fine-tuned for 30 epochs to recover potential performance degradation caused by pruning. Fine-tuning was conducted using the same training dataset, optimizer, loss function, and learning-rate schedule as those used for the baseline MobileNetV3-Large model. The resulting models were subsequently evaluated in terms of classification accuracy, parameter count, FLOPs, and inference latency.

### 3.1 Comparison of classification performance among different backbones

**Figure 3:**
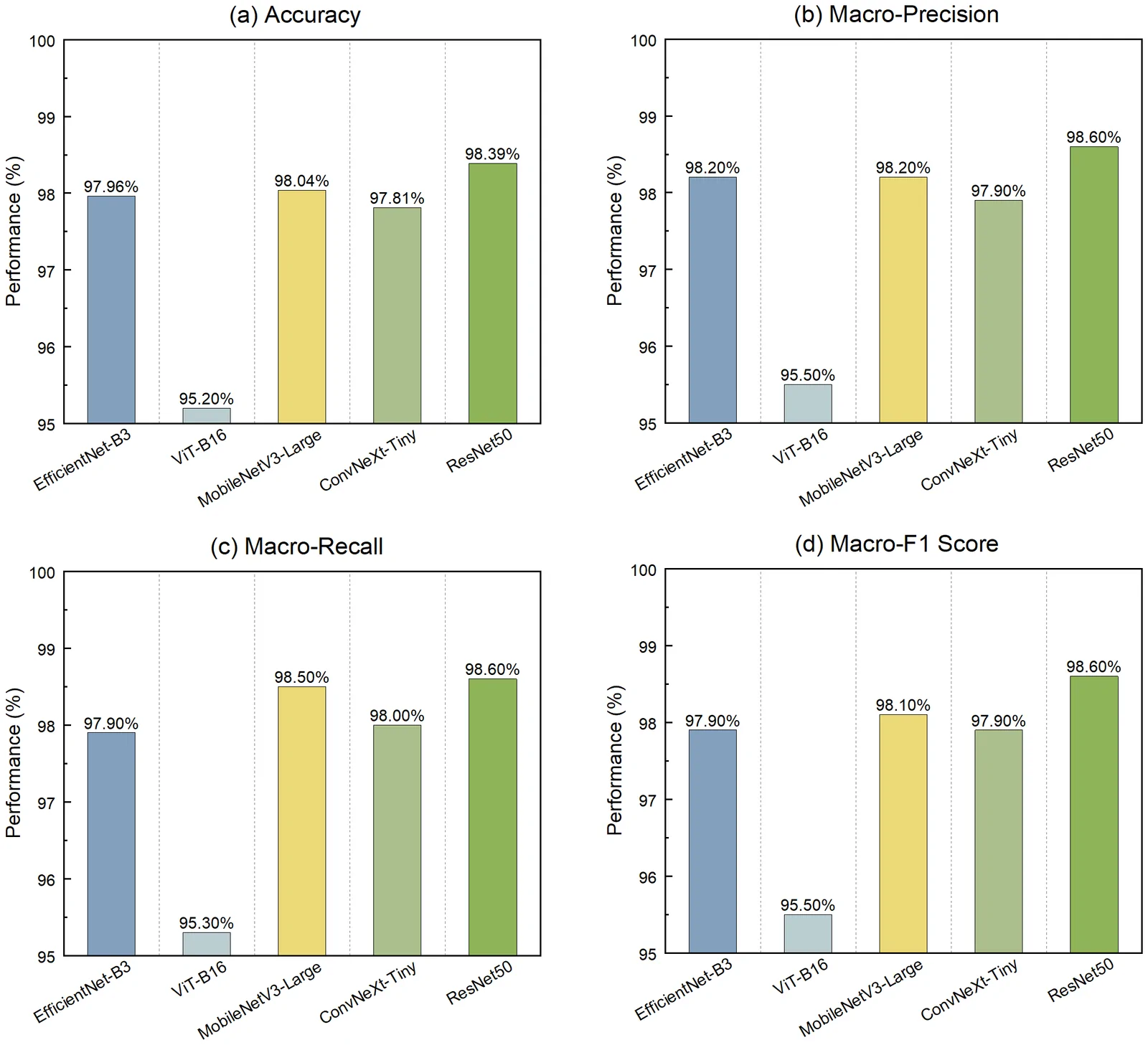
Comparison of classification performance among five deep learning models for tea cultivar identification. **(a)** Accuracy, **(b)** macro-precision, **(c)** macro-recall, and **(d)** macro-F1 score.

After data augmentation, the training dataset was expanded to 8,885 images, while validation and testing sets remained unchanged. Five representative backbone models, including ConvNeXt-Tiny, EfficientNet-B3, MobileNetV3-Large, ResNet50, and ViT-B16, were evaluated using accuracy, macro-precision, macro-recall, macro-F1 score, and confusion matrices (**Figure 3**).

ResNet50 achieved the best classification performance, with an accuracy of 98.39% and macro-F1 score of 98.6%. MobileNetV3-Large showed comparable performance (accuracy: 98.04%, macro-F1: 98.1%) while maintaining a lightweight architecture suitable for mobile deployment. In contrast, ViT-B16 achieved relatively lower performance (accuracy: 95.2%), indicating that pure transformer-based architectures may require larger datasets to fully exploit their representation capability (**Supplementary Table 4**).

Overall, all evaluated models achieved classification accuracies above 95%, confirming the effectiveness of deep visual representation learning for tea cultivar identification. All experiments were conducted under identical experimental settings with a fixed random seed (42) to ensure reproducibility. Although ResNet50 achieved slightly higher classification performance, MobileNetV3-Large was selected as the baseline model due to its favorable trade-off between classification accuracy, computational efficiency, and mobile deployment suitability.

To further facilitate quantitative comparison across individual tea cultivars, the class-wise precision, recall, and F1-score achieved by the baseline MobileNetV3-Large model on the held-out test set are provided in **Supplementary Table 3**.

### 3.2 Confusion matrix analysis of different deep learning backbones

**Figure 4:**
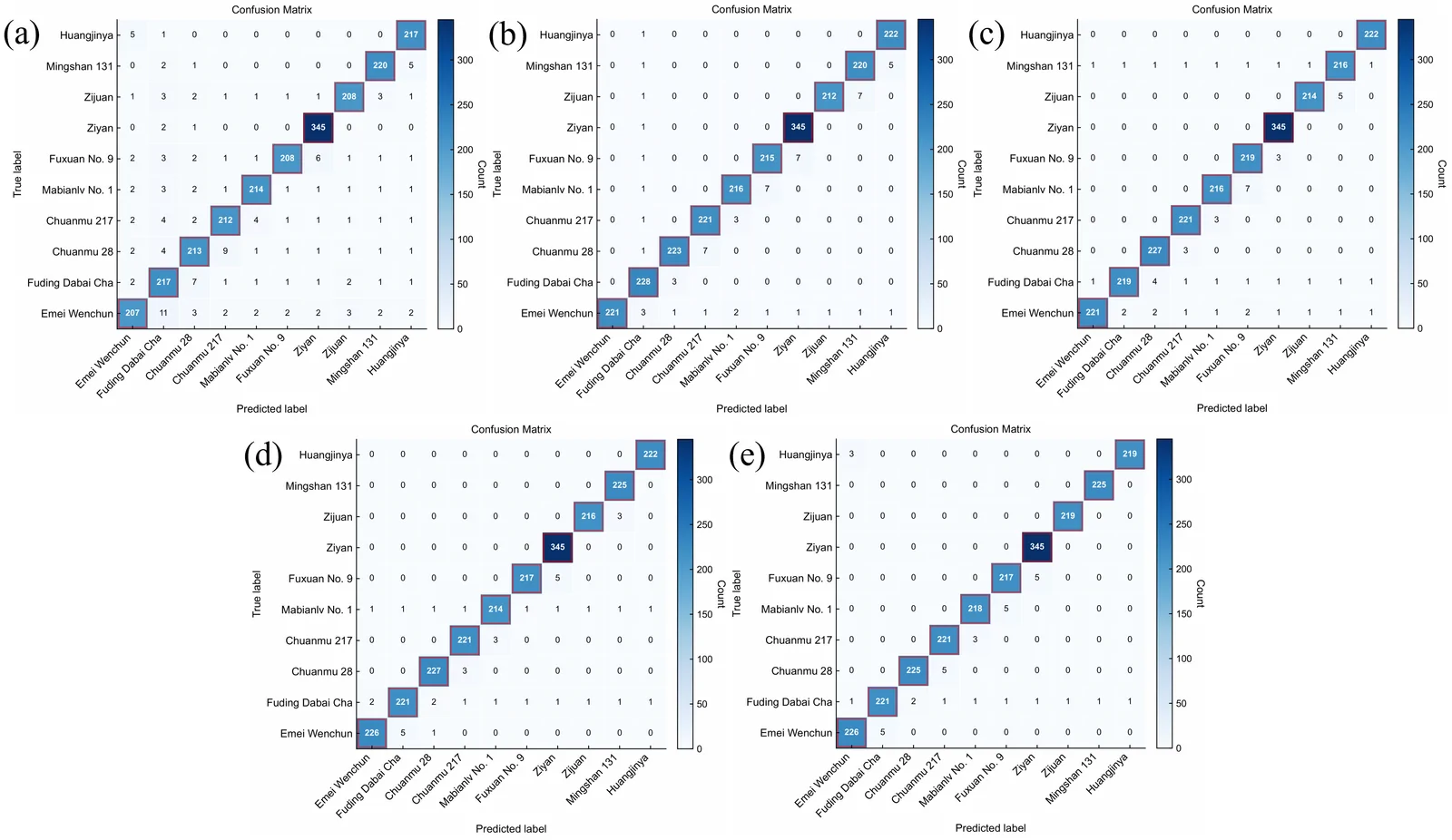
Confusion matrices of different deep learning models for tea cultivar classification. Results are shown for **(a)** ViT-B16, **(b)** ConvNeXt-Tiny, **(c)** EfficientNet-B3, **(d)** MobileNetV3-Large, and **(e)** ResNet50.

To further investigate the class-wise classification performance of different backbone models, confusion matrices were analyzed for all five architectures (**Figure 4**). The dominant diagonal elements in all confusion matrices indicate that most tea cultivar categories were correctly recognized, demonstrating the effectiveness of deep learning models for fine-grained cultivar identification.

ResNet50 and MobileNetV3-Large exhibited more concentrated diagonal distributions, consistent with their superior overall performance reported in Section 3.1. Several cultivars, including ‘Ziyan’ and ‘Zijuan’, achieved near-perfect recognition across different models, whereas misclassifications mainly occurred among visually similar cultivars. For example, ‘Chuanmu 217’ and ‘Chuanmu 28’ showed relatively higher mutual confusion, likely due to their similar leaf morphology and vein characteristics. Confusion between ‘Fuxuan No. 9’ and ‘Huangjinya’ was also observed, indicating the challenge of distinguishing cultivars with subtle phenotypic differences.

### 3.3 Selection and training analysis of MobileNetV3-large baseline

**Figure 5:**
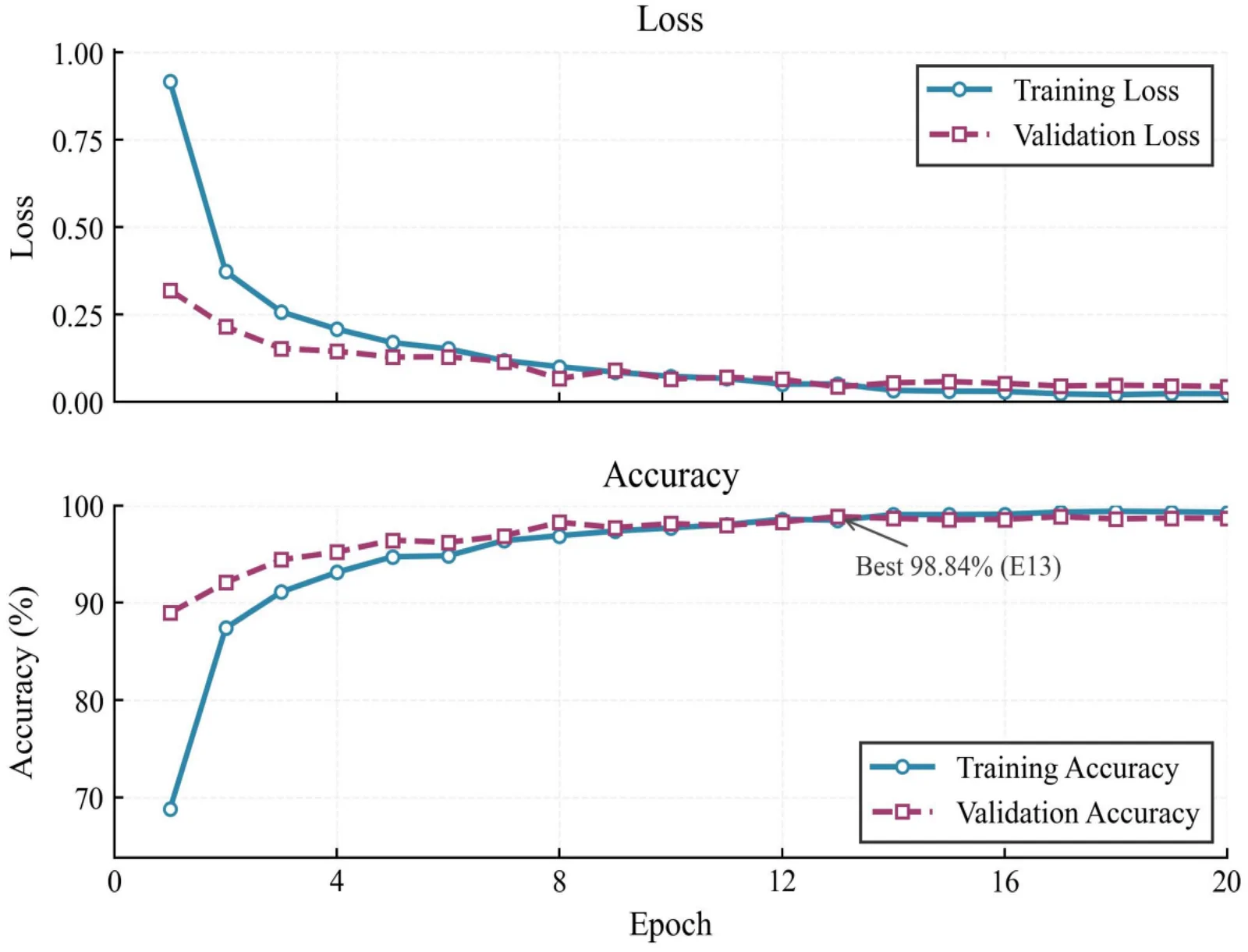
Training and validation curves of the lightweight tea cultivar identification model, showing loss and accuracy changes over epochs.

Based on the comparative results in Section 3.1, MobileNetV3-Large was selected as the baseline model for subsequent lightweight optimization due to its favorable trade-off between classification performance and deployment efficiency. To further evaluate the optimization stability of the selected baseline model, the training and validation curves were analyzed (**Figure 5**).

The training process exhibited rapid convergence during the early epochs, with both training and validation losses decreasing continuously and gradually stabilizing thereafter. The close agreement between training and validation curves indicates that the model achieved stable optimization without evident overfitting. The training and validation accuracies remained consistent throughout the training process, with the highest validation accuracy reaching 98.84% at epoch 13. The final training accuracy (99.28%) showed only a small gap compared with the validation accuracy, suggesting reliable generalization capability under the current dataset. Considering its high recognition accuracy, stable convergence behavior, and suitability for resource-constrained deployment, MobileNetV3-Large was selected as the baseline model for subsequent pruning, quantization, and mobile deployment experiments.

### 3.4 Channel pruning strategy and performance evaluation

#### 3.4.1 Experimental configuration of channel pruning strategies

Following the structured channel-pruning procedure described in Section 2.7, L1-norm-based pruning, biological feature-guided pruning (BIO), and the proposed FUSED strategy were evaluated on the selected MobileNetV3-Large baseline. Pruning was applied to the eligible inverted residual blocks, while the stem convolution and final classifier were excluded. Detailed implementation settings are provided in **Supplementary Table 2**.

To ensure a fair comparison in the ablation study, all pruning variants were evaluated at a fixed pruning rate of 20%. Consequently, the pruned models had the same computational complexity, with 4.44 M parameters and 0.61 G FLOPs. After channel removal, each model was fine-tuned for 30 epochs using the same training dataset, optimizer, loss function, and learning-rate schedule as the baseline model.

The contributions of the L1, BIO, and SE-based importance criteria were first examined through ablation experiments, as presented in Section 3.4.2. Based on the optimal channel-ranking strategy identified in the ablation study, pruning rates ranging from 10% to 40% were subsequently evaluated to investigate the trade-off among classification performance, model complexity, and inference efficiency in Section 3.4.3.

#### 3.4.2 Ablation analysis of the FUSED channel pruning strategy

To evaluate the contribution of each channel importance criterion, an ablation study was conducted by progressively combining the L1, BIO, and SE-based criteria(**Table 3**). For a fair comparison, all pruned variants were evaluated at a fixed pruning rate of 20%, resulting in the same model complexity of 4.44 M parameters and 0.61 G FLOPs.

**Table 3:** Ablation analysis of channel importance criteria in the FUSED pruning strategy.

Model	L1 criterion	BIO criterion	SE module	Parameters (M)	FLOPs (G)	Top-1 (%)	Top-3 (%)	Top-1 vs. L1-only (percentage points)
Baseline	–	–	–	5.49	0.92	98.84	100	N/A
L1 pruning	✓	–	–	4.44	0.61	96.85	99.87	0.00
BIO pruning	–	✓	–	4.44	0.61	96.39	99.82	-0.46
L1+BIO	✓	✓	–	4,44	0.61	97.00	99.78	+0.15
FUSED	✓	✓	✓	4.44	0.61	97.50	99.96	+0.65

N/A indicates that the unpruned baseline was not included in the relative comparison among pruning strategies.

As shown in **Table 3**, L1-only pruning achieved a Top-1 accuracy of 96.85%, whereas BIO-only pruning achieved 96.39%. Combining the L1 and BIO criteria increased Top-1 accuracy to 97.00%, representing an improvement of 0.15 percentage points over L1-only pruning. After incorporating the SE attention score, the complete FUSED strategy achieved the highest Top-1 accuracy of 97.50%, which was 0.65 percentage points higher than that of L1-only pruning.

The FUSED strategy also achieved a Top-3 accuracy of 99.96%, approaching the performance of the unpruned baseline. These results indicate that structural weight magnitude, tea-leaf-specific morphological responses, and SE-derived channel attention provide complementary information for channel importance estimation. Therefore, the complete FUSED strategy was adopted for the subsequent pruning-rate experiments.

#### 3.4.3 Effect of pruning rates on model complexity and classification performance

Based on the ablation results, the complete FUSED strategy was used to evaluate the effects of different pruning rates on model complexity, classification performance, and inference latency.

**Figure 6:**
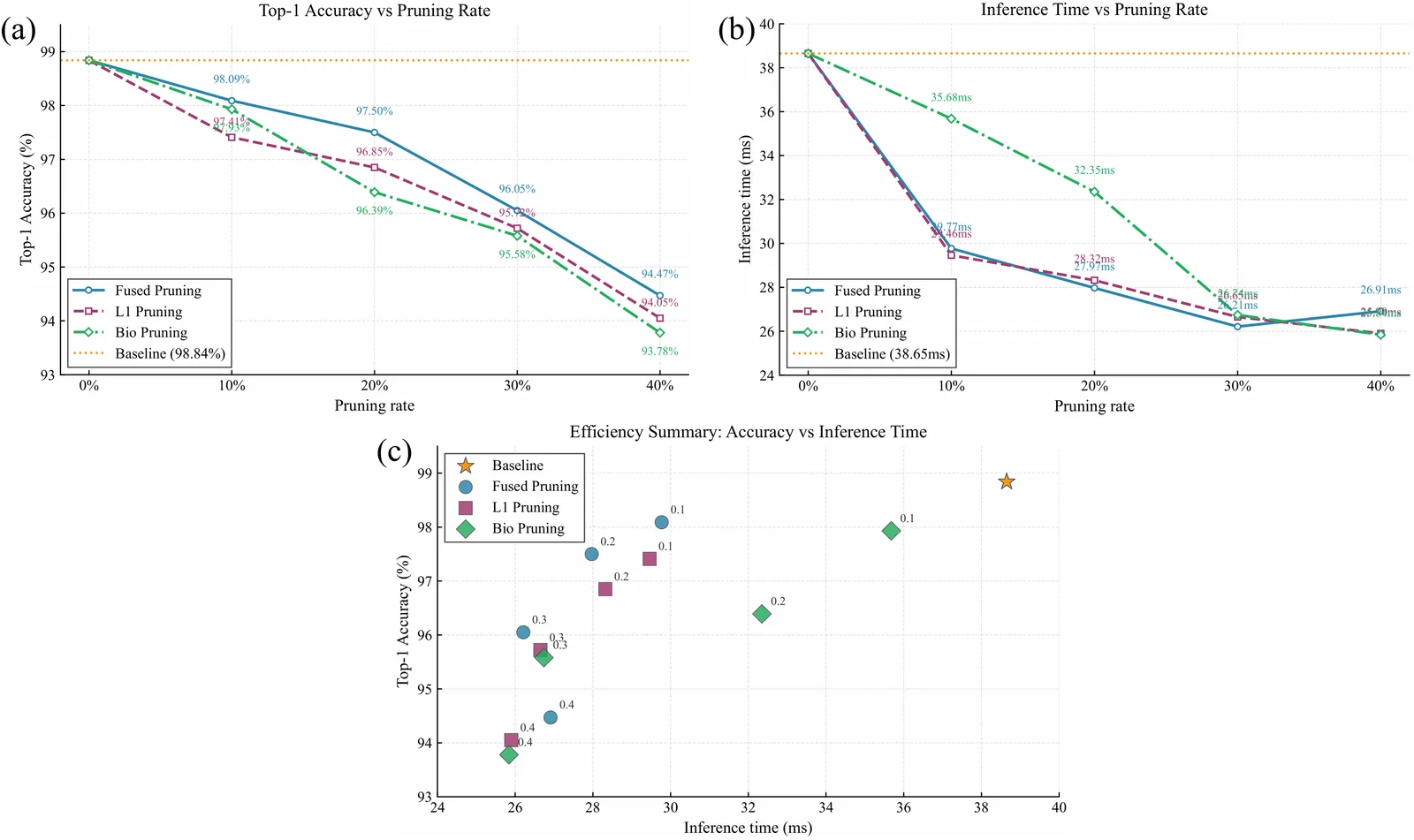
Performance comparison of channel-pruning strategies for lightweight tea-cultivar classification. **(a)** Top-1 accuracy at different pruning rates. **(b)** Inference latency at different pruning rates. **(c)** Accuracy–latency trade-off for FUSED, L1, and BIO pruning.

As shown in **Figures 6a**, Top-1 accuracy gradually decreased as the pruning rate increased. Nevertheless, the FUSED strategy consistently maintained higher classification accuracy than the individual L1 and BIO strategies at the same pruning rate. Meanwhile, increasing the pruning rate reduced inference latency, although the reduction was not strictly proportional to the pruning ratio (**Figure 6b**). The accuracy–latency trade-off shown in **Figure 6c** further demonstrates that FUSED pruning provided a more favorable balance between classification performance and inference efficiency.

The complete numerical results are provided in **Supplementary Table 5**. As the pruning rate increased from 0% to 40%, the number of parameters decreased from 5.49 M to 3.31 M, and FLOPs decreased from 0.92 G to 0.39 G. Meanwhile, Top-1 accuracy gradually declined from 98.84% to 94.47%. At a pruning rate of 20%, the model retained a Top-1 accuracy of 97.50%, while reducing the number of parameters and FLOPs to 4.44 M and 0.61 G, respectively. Therefore, the 20% pruning rate was selected as the final configuration because it provided a favorable trade-off between classification performance and computational complexity.

### 3.5 Quantization, deployment efficiency, and interpretability

#### 3.5.1 PTDQ and deployment efficiency

Following channel pruning optimization, PTDQ (PTDQ) was applied to further reduce model storage and inference overhead. The implementation details of the quantization procedure are provided in **Supplementary Table 6**. The weights of convolutional and fully connected layers were quantized from FP32 to INT8 representation, while activation values were dynamically quantized during inference. The quantization process was implemented using the PyTorch quantization framework.

**Figure 7:**
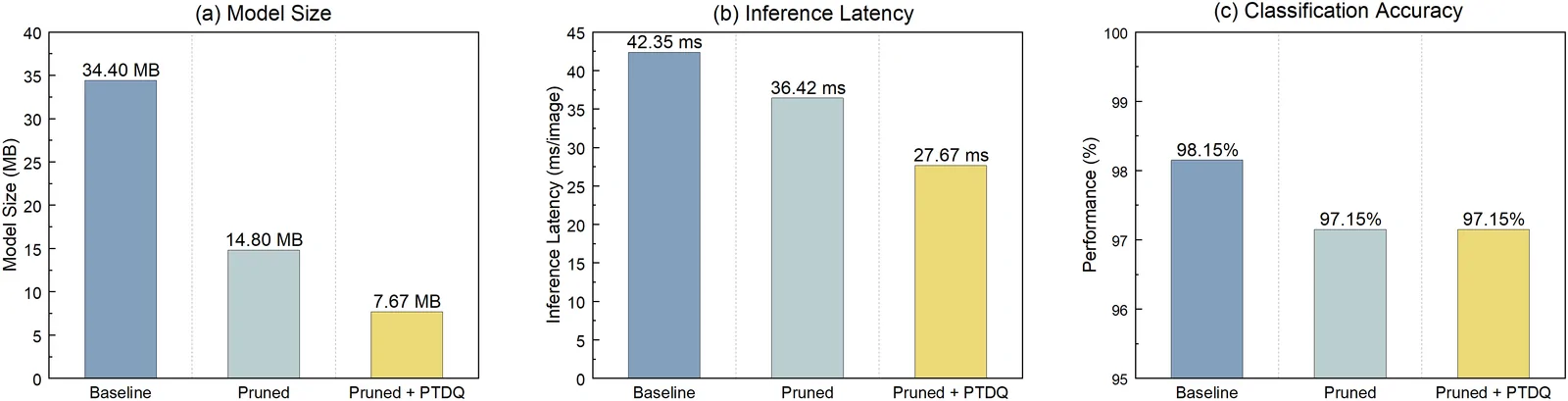
Effects of channel pruning and PTDQ **(a)** Comparison of model size before and after lightweight optimization. **(b)** Comparison of inference latency before and after lightweight optimization. **(c)** Comparison of classification accuracy before and after lightweight optimization.

The effects of channel pruning and PTDQ (PTDQ) on model size, inference latency, and classification accuracy are shown in **Figures 7a-c**. Compared with the baseline MobileNetV3-Large model, channel pruning reduced the model size from 34.40 MB to 14.80 MB and decreased the inference latency from 42.35 ms/image to 36.42 ms/image. After PTDQ, the model size was further reduced to 7.67 MB, while the inference latency decreased to 27.67 ms/image.

As shown in **Figure 7c**, channel pruning resulted in a slight reduction in test-set accuracy, whereas subsequent PTDQ introduced no additional performance degradation. The detailed accuracy values corresponding to different experimental stages, including baseline training evaluation, backbone comparison, pruning optimization, and PTDQ deployment evaluation, are provided in **Supplementary Table 6**. Overall, the combination of structured channel pruning and PTDQ achieved a favorable balance between classification performance and deployment efficiency, demonstrating its potential for resource-constrained applications.

To quantify the individual contribution of pruning and quantization, an ablation experiment was conducted (**Table 4**). Pruning reduced the model size from 34.40 MB to 14.80 MB and latency from 43.25 ms to 36.42 ms, while maintaining a Top-1 accuracy of 97.15%. Applying INT8 quantization further reduced the model size to 7.67 MB and latency to 27.67 ms without additional accuracy degradation.

**Table 4:** Ablation analysis of channel pruning and INT8 quantization.

Model	Precision	Size (MB)	Latency (ms)	Top-1
Baseline	FP32	34.40	43.25	98.15
Pruned	FP32	14.80	36.42	97.15
Pruned+INT8	INT8	7.67	27.67	97.15

#### 3.5.2 Feature preservation and interpretability analysis

To investigate whether model compression affects feature representation and discriminative ability, t-SNE visualization. Grad-CAM analysis was performed on the original and optimized MobileNetV3-Large models using the last convolutional feature extraction layer before the classifier.

**Figure 8:**
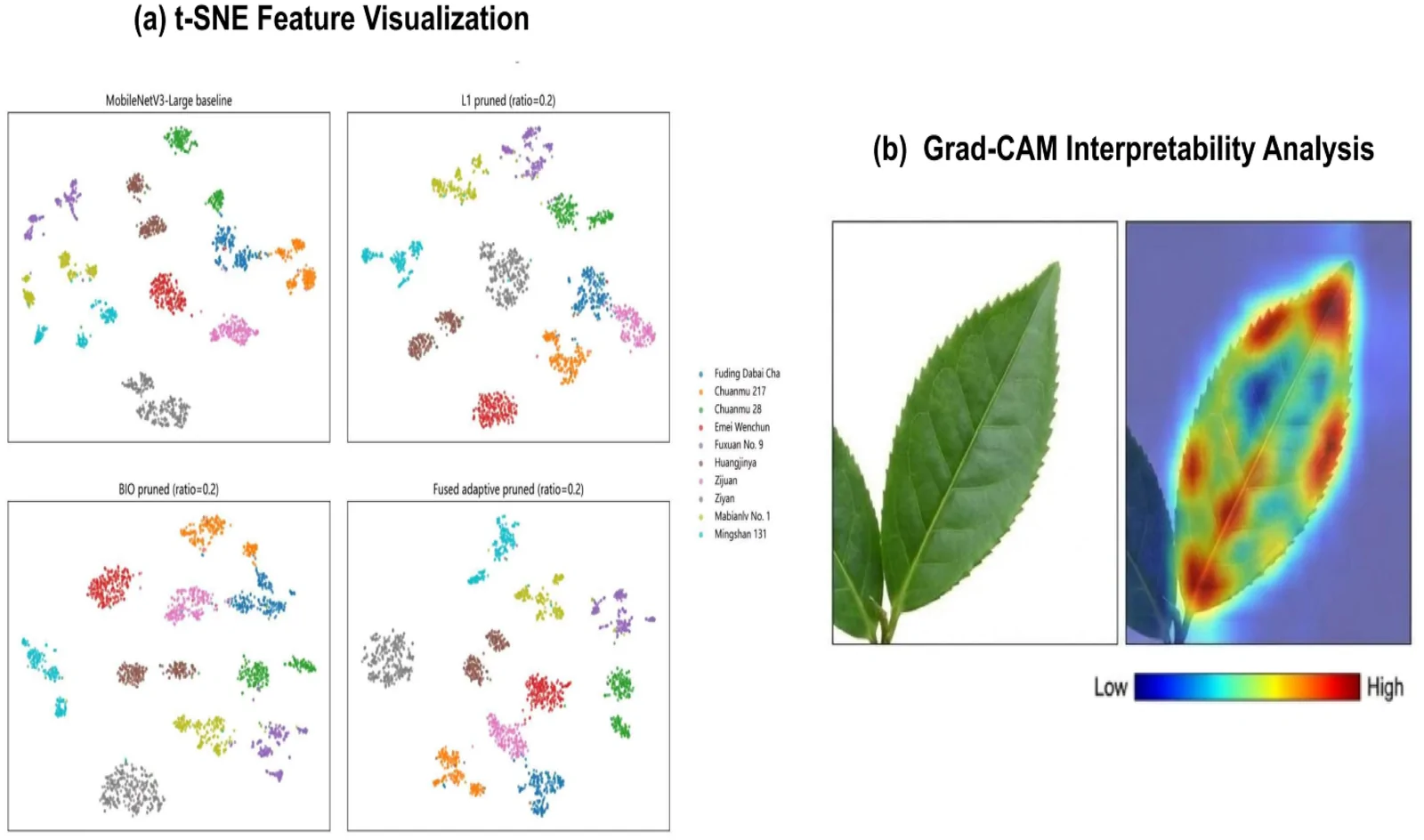
Visualization of feature representations and model attention after lightweight optimization. **(a)** t-SNE plots showing feature distributions of different MobileNetV3-Large variants after pruning. **(b)** Grad-CAM visualization of the optimized model highlighting image regions associated with tea cultivar identification.

As shown in **Figure 8a**, the baseline MobileNetV3-Large model produced well-separated feature clusters for different tea cultivar categories. After pruning, the L1 and BIO pruning strategies caused slight variations in feature distribution, with increased intra-class dispersion or reduced inter-class separation in some categories. In contrast, the FUSED pruning strategy maintained feature clustering patterns comparable to the original model, indicating that the proposed pruning strategy effectively preserved discriminative representations after channel reduction.

Furthermore, Grad-CAM visualization was conducted to evaluate whether the optimized model retained meaningful spatial attention regions. As shown in **Figure 8b**, the compressed model mainly focused on biologically relevant leaf regions, including the midrib, leaf edges, and texture-rich areas, rather than background regions. These results provide qualitative evidence that the pruning and quantization procedures preserved the essential visual cues required for tea cultivar identification.

## 4 Discussion

### 4.1 Feasibility of lightweight framework for leaf image-based tea cultivar identification

Conventional image-processing and early machine-learning approaches for tea cultivar identification often rely on handcrafted features and may show limited robustness under complex field conditions. Deep learning has substantially improved automatic feature extraction and recognition performance. For example, Ding et al. evaluated five lightweight CNNs using leaf images, but their study was limited to three cultivars and approximately 1,800 images, potentially constraining model generalizability (Ding et al., 2023). Zhang et al. expanded the task to 16 cultivars using DenseNet201 and field canopy images (Zhang et al., 2025b). However, canopy-based recognition is susceptible to complex visual interference, while parameter-intensive networks may impose substantial computational and storage demands.

To achieve reliable tea cultivar identification under complex field conditions, selecting an appropriate plant organ as the recognition target is critical. Previous studies have largely relied on field-acquired tea canopy images or seasonally available tender shoots for identification (Cao et al., 2023; Zhang et al., 2025b). However, visual recognition under such conditions is often challenged by overlapping branches, cluttered backgrounds, and image degradation caused by wind-induced shoot motion. In addition, tender shoots exhibit substantial variations in size, growth orientation, and leaf unfolding angle, further increasing the difficulty of accurate visual recognition (Wang et al., 2025). In contrast, fully expanded mature leaves exhibit well-developed and readily quantifiable morphological characteristics, including leaf area, perimeter, length, width, and aspect ratio. These traits have been shown to capture phenotypic variation among tea accessions with diverse genetic backgrounds and can therefore serve as informative phenotypic descriptors for tea cultivar differentiation (Hodac et al., 2024; Jiang et al., 2026). Therefore, mature leaves provide a readily obtainable and phenotypically informative basis for tea cultivar identification.

Model quantization has been widely recognized as an effective strategy for reducing computational complexity and memory requirements while maintaining competitive predictive performance. Tasci et al. demonstrated that a quantized LeNet-5 achieved 99.87% classification accuracy with only 23.1 KB of parameter storage and substantially reduced computational cost, highlighting the suitability of quantized models for resource-constrained edge devices (Tasci et al., 2023). (Furthermore, Wang et al. showed that combining channel pruning with quantization could further improve deployment efficiency in plant disease recognition while maintaining high classification accuracy (Wang et al., 2021). Although these studies primarily focused on conventional CNN architectures, they collectively demonstrate the effectiveness of explicit model compression for agricultural vision tasks. Building on these findings, the present study further compressed an inherently efficient MobileNetV3-Large backbone using Fused channel pruning and PTDQ. Compared with the unoptimized baseline model, the proposed compression pipeline reduced model size from 34.40 MB to 7.67 MB and decreased inference latency from 43.25 ms to 27.67 ms, while maintaining competitive classification accuracy (98.15% to 97.15%). Therefore, additional compression can still provide practical deployment benefits even for an already lightweight architecture.

The mechanism underlying this efficiency improvement was further supported by the Fused pruning strategy, which integrates multiple channel importance criteria to reduce redundant parameters. Although pruning introduced a slight decrease in classification accuracy, the optimized models maintained clear class separation in the t-SNE feature space, suggesting that lightweight optimization did not substantially disrupt discriminative feature representations. Furthermore, the architectural advantages of MobileNetV3-Large, including depthwise separable convolutions and squeeze-and-excitation modules, contribute to efficient feature extraction with reduced computational complexity. These characteristics make MobileNetV3-Large suitable for tea cultivar identification scenarios requiring both accuracy and deployment efficiency.

Grad-CAM visualizations reveal that the model focuses on biologically relevant regions, including the midrib, lateral veins, and serrated margins, which are consistent with traditional morphological criteria for cultivar discrimination (UPOV, 2022). This suggests that the model captures interpretable and phenotypically meaningful features, supporting its application in high-throughput tea cultivar identification.

### 4.2 Limitations and prospect

Despite these promising results, several limitations remain to be addressed in future work. First, while the 10 clonal tea cultivars evaluated in this study present distinct leaf characteristics(**Table 1**), China has registered over 300 national and provincial elite tea cultivars. Many of these closely related cultivars share highly similar leaf and canopy phenotypes, posing a substantial challenge for fine-grained image-based recognition (Chen et al., 2022a). Consequently, the robustness of our model in distinguishing morphologically analogous cultivars must be further validated in future work by broadening the cultivar scope and enriching the dataset with visually similar samples (Sun et al., 2023).

Second, the current dataset mainly consists of healthy, fully expanded leaves collected from mature tea plants. However, in practical propagation, nursery management, and plant variety protection, tea cultivar identification may also be required for juvenile clonal plants produced from cuttings (Huang et al., 2016; UPOV, 2022). Because tea leaf phenotypes may vary with plant age, leaf developmental position, and growing conditions, it remains unclear whether the discriminative features learned from mature plants are consistent in juvenile nursery plants and whether such stage- and environment-related differences would significantly affect identification accuracy (Wang et al., 2016a; Zhang et al., 2020; Chen et al., 2022b).

Finally, the optimized lightweight model provides a robust foundation for mobile-assisted applications. Real-device deployment, energy consumption analysis, and large-scale field validation under diverse agricultural scenarios should be explored in future studies to fully realize its practical potential.

This study developed a lightweight deep learning-based framework for non-destructive tea cultivar identification using mature leaf images. A dataset containing 11,404 original leaf images from 10 cultivars was constructed, with augmentation applied only to the training set. Among five representative deep learning architectures, MobileNetV3-Large provided a favorable balance between classification accuracy and computational efficiency. Through pruning and PTDQ, the model size was reduced from 34.40 MB to 7.67 MB, while inference latency decreased from 43.25 ms to 27.67 ms with only a slight reduction in classification accuracy (98.15% to 97.15%). These results demonstrate the potential of lightweight deep learning models for efficient tea cultivar identification under resource-constrained conditions.

## Data Availability

The raw data supporting the conclusions of this article will be made available by the authors, without undue reservation.
