# Supplementary material for: A lightweight deep learning framework for tea cultivar identification based on leaf images
Source: Front Plant Sci. 2026 Aug 20;17:1863075. doi: 10.3389/fpls.2026.1863075 (PMC13538894; doi:10.3389/fpls.2026.1863075)
Supplement: Supplementary file 1 [file DataSheet1.pdf]

Supplementary Material

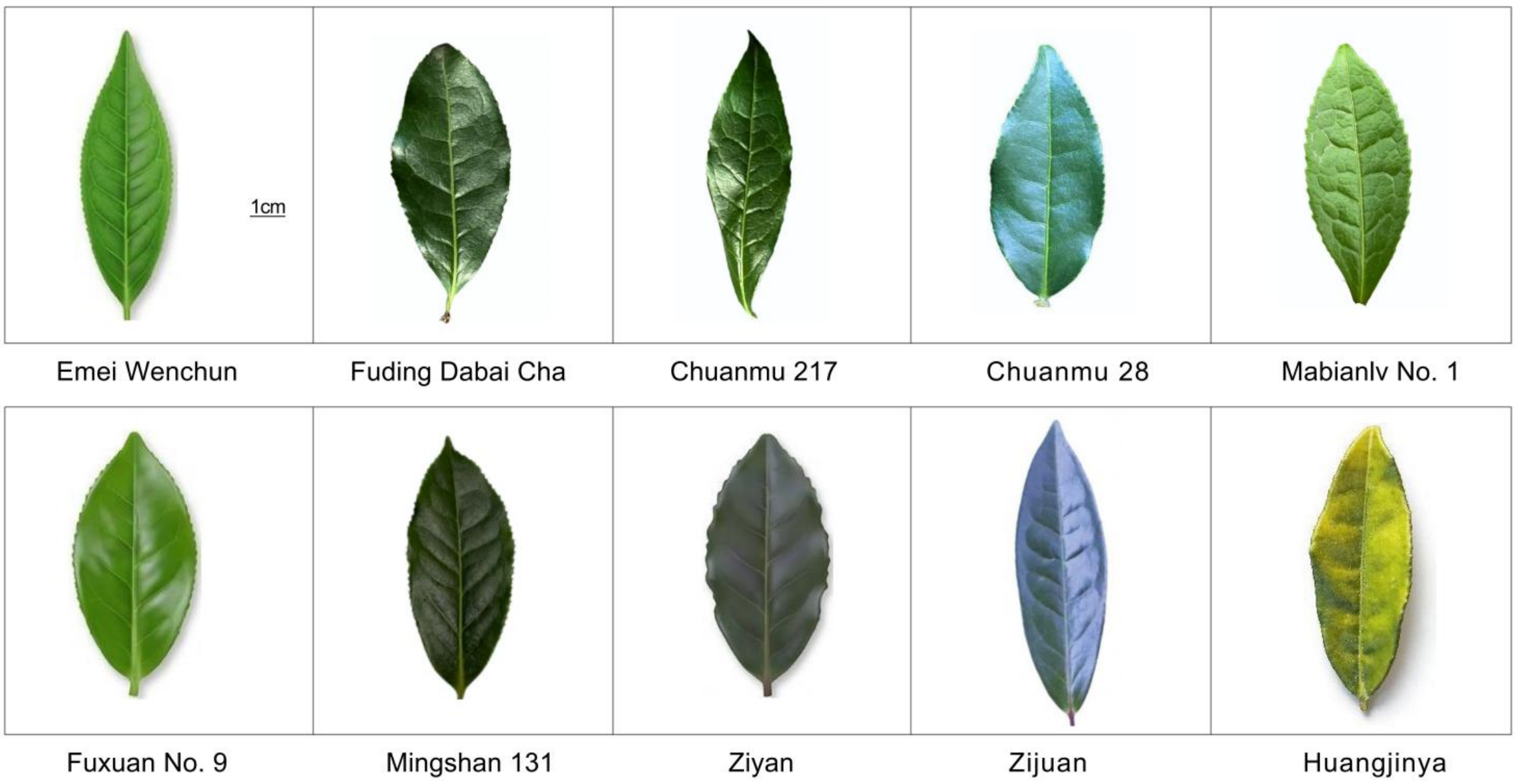

Supplementary Figure 1. Comparative morphology of leaves from ten different tea plant cultivars.

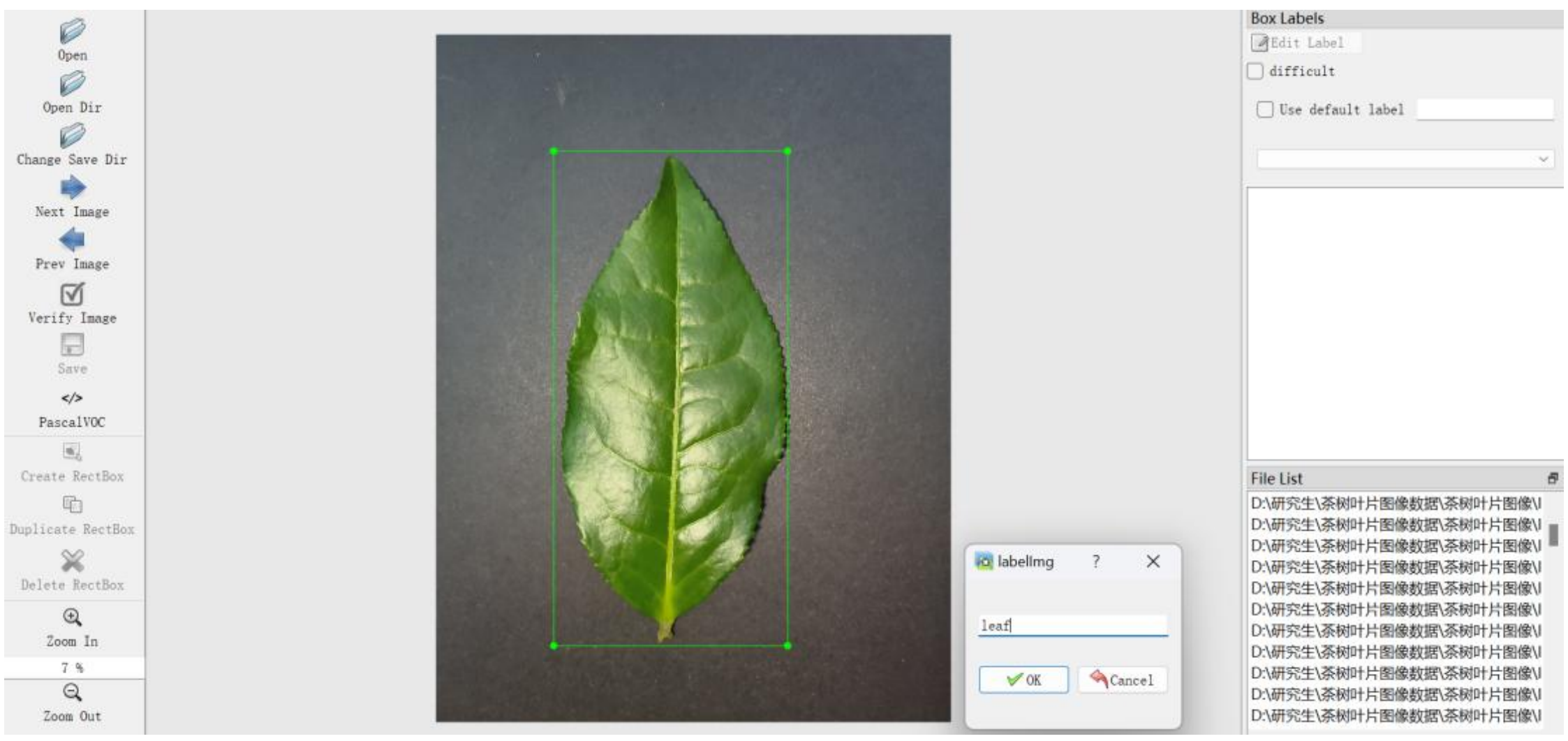

Supplementary Figure 2. Example of manual leaf ROI annotation using LabelImg.

Supplementary Table 1. Training hyperparameters and experimental settings for backbone comparison.

Parameter	Setting
Random seed	42
Batch size	32
Epochs	20
Optimizer	Adam
Gradient clipping	Applied
Initial learning rate	2.4×10^{-4}
Weight decay	1.0×10^{-5}
Input resolution	448 × 448 × 3
Learning rate scheduler	Cosine annealing
Loss function	Cross-entropy loss
Training dataset	Same training split
Initialization	ImageNet pretrained
Model selection	Best validation accuracy
Evaluation protocol	Accuracy, precision, recall, F1-score
Data augmentation	Random rotation, horizontal flipping, scaling (crop ratio: 0.8 - 1.0), contrast adjustment, hue shifting, Gaussian noise, random occlusion, and motion blur.

Supplementary Table 2. Implementation details of structured channel pruning.

Item	Setting
Pruning type	Structured channel pruning
Importance criteria	L1, BIO, and SE attention
Granularity	Channel
Fusion strategy	weighted combination
Fusion weights	w1=0.2,w2=0.5,w3=0.3
Pruning rate used for ablation:	20%
Fine-tuning epochs	30
Pruned layers	MobileNetV3 inverted residual blocks
Excluded layers	First convolution and classifier
Channel dimension	Output channels

Supplementary Table 3. Comparison of classification performance across candidate architectures.

Model	Accuracy	Precision	Recall	F1-score
EfficientNet-B3	0.9796	0.982	0.979	0.979
ViT-B16	0.952	0.955	0.953	0.955
MobileNetV3-large	0.9804	0.982	0.985	0.981
ConvNeXt-Tiny	0.9781	0.979	0.980	0.979
ResNet50	0.9839	0.986	0.986	0.986

Supplementary Table 4. Class-wise precision, recall, and F1-score of the baseline MobileNetV3-Large model evaluated on the independent test set.

Cultivar	Precision (%)	Recall (%)	F1-score (%)
Fuding Dabai Cha	97.00	96.00	96.00
Chuanmu 217	98.00	99.00	98.00
Chuanmu 28	96.00	99.00	97.00
Emei Wenchun	96.00	98.00	97.00
Fuxuan No.9	98.00	98.00	98.00
Huangjinya	100.00	100.00	100.00
Zijuan	100.00	99.00	99.00
Ziyan	100.00	100.00	100.00
Mabianlv No.1	98.00	96.00	97.00
Mingshan 131	99.00	100.00	99.00

Supplementary Table 5. Effect of pruning ratio on model complexity, classification performance, and inference efficiency.

Pruning rate	Parameters(M)	FLOPs(G)	Top-1(%)	Latency(ms)
0%	5.49	0.92	98.84	38.65
10%	4.95	0.78	98.09	30.47
20%	4.44	0.61	97.50	28.32
30%	3.86	0.50	96.05	26.21
40%	3.31	0.39	94.47	25.80

Supplementary Table 6. Implementation details of PTDQ.

Component	Precision
Convolution weights	INT8
Fully connected weights	INT8
Intermediate activation	Dynamic INT8
Activation	FP32/INT8
Bias	FP32
Framework	PyTorch

Supplementary Table 7. Summary of MobileNetV3-Large performance at different experimental stages

Experimental stage	Dataset split	Purpose	Accuracy
Training monitoring	Validation	Best epoch selection	98.84%
Backbone comparison	Test	Architecture comparison	98.04%
Channel pruning	Test	Compression evaluation	97.15%
Pruning + PTDQ	Test	Deployment evaluation	97.15%
